# The Transcriptome of *Cunninghamia lanceolata* male/female cone reveal the association between MIKC MADS-box genes and reproductive organs development

**DOI:** 10.1186/s12870-020-02634-7

**Published:** 2020-11-05

**Authors:** Dandan Wang, Zhaodong Hao, Xiaofei Long, Zhanjun Wang, Xueyan Zheng, Daiquan Ye, Ye Peng, Weihuang Wu, Xiangyang Hu, Guibin Wang, Renhua Zheng, Jisen Shi, Jinhui Chen

**Affiliations:** 1 Key Laboratory of Forestry Genetics & Biotechnology of Ministry of Education, Co-Innovation Center for Sustainable Forestry in Southern China, Nanjing Forestry University, Nanjing, 210037 China; 2 Fujian Academy of Forestry, Fuzhou, 350012 China; 3 College of Life Sciences, Hefei Normal University, Hefei, 230601 China; 4 National Germplasm Bank of Chinese fir at Fujian Yangkou Forest Farm, Shunchang, 353211 China; 5 College of Biology and the Environment, Nanjing Forestry University, Nanjing, 210037 China; 6 Shanghai Key Laboratory of Bio-Energy Crops, School of Life Sciences, Shanghai University, Shanghai, 200444 China; 7 Co-Innovation Center for Sustainable Forestry in Southern China, Nanjing Forestry University, Nanjing, 210037 China

**Keywords:** Cone development, *C. lanceolata*, transcriptome, MADS-box gene, Floral development model

## Abstract

**Background:**

*Cunninghamia lanceolata* (Chinese fir), a member of the conifer family Cupressaceae, is one of the most popular cultivated trees for wood production in China. Continuous research is being performed to improve *C. lanceolata* breeding values. Given the high rate of seed abortion (one of the reasons being the failure of ovule and pollen development) in *C. lanceolata*, the proper formation of female/male cones could theoretically increase the number of offspring in future generations. MIKC MADS-box genes are well-known for their roles in the flower/cone development and comprise the typical/atypical floral development model for both angiosperms and gymnosperms.

**Results:**

We performed a transcriptomic analysis to find genes differentially expressed between female and male cones at a single, carefully determined developmental stage, focusing on the MIKC MADS-box genes. We finally obtained 47 unique MIKC MADS-box genes from *C. lanceolata* and divided these genes into separate branches. 27 out of the 47 MIKC MADS-box genes showed differential expression between female and male cones, and most of them were not expressed in leaves. Out of these 27 genes, most B-class genes (*AP3/PI*) were up-regulated in the male cone, while *TM8* genes were up-regulated in the female cone. Then, with no obvious overall preference for *AG* (class C + D) genes in female/male cones, it seems likely that these genes are involved in the development of both cones. Finally, a small number of genes such as *GGM7*, *SVP*, *AGL15*, that were specifically expressed in female/male cones, making them candidate genes for sex-specific cone development.

**Conclusions:**

Our study identified a number of MIKC MADS-box genes showing differential expression between female and male cones in *C. lanceolata*, illustrating a potential link of these genes with *C. lanceolata* cone development. On the basis of this, we postulated a possible cone development model for *C. lanceolata*. The gene expression library showing differential expression between female and male cones shown here, can be used to discover unknown regulatory networks related to sex-specific cone development in the future.

## Background

**Fig. 1:**
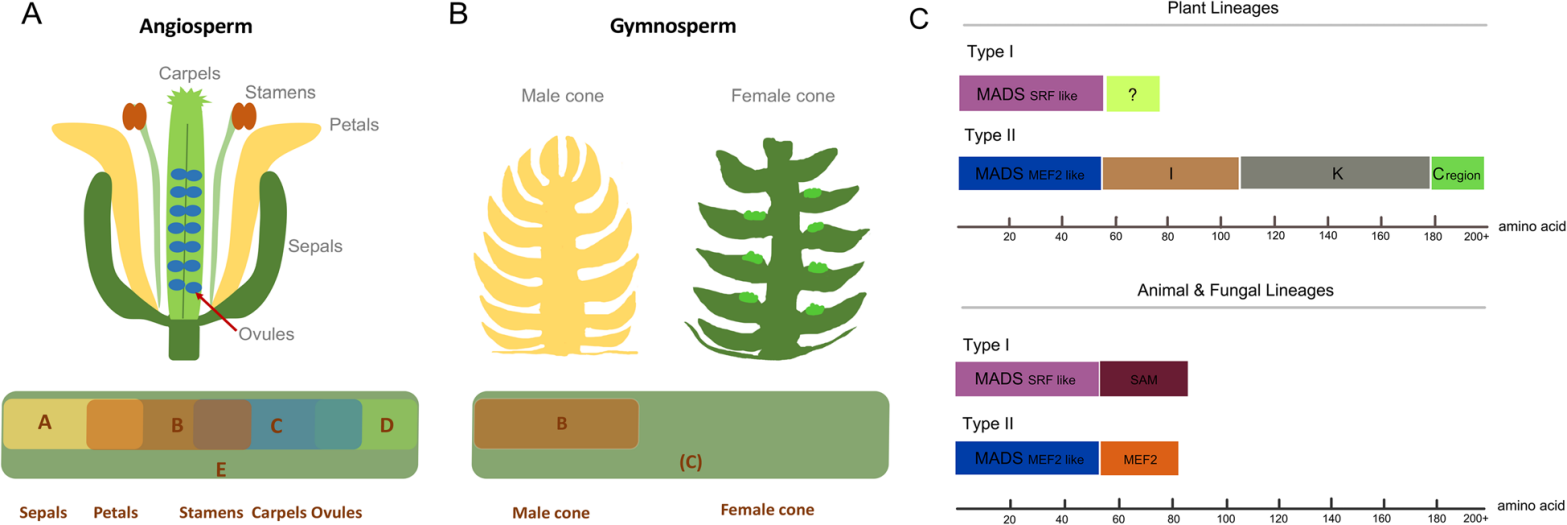
Floral homeotic functions in angiosperms and gymnosperms and MADS-box gene domains in different species. **a** Diagram illustrating the classic ABCDE floral development model in the angiosperm *Arabidopsis thaliana*. Different combinations of A, B, C, D and E classes lead to different organ identity [4]. **b** B(C) model was proposed to control the development of male and female cones in gymnosperms, (C) indicates C + D [4–6]. **c** Two type of MADS-box proteins are shown: type I (SRF-like) and type II (MEF2-like). “The scale indicates the number of amino acids of the protein. The “?” indicates that the C-terminal is not well defined yet” [7] (redrawn from Fig. 1(https://www.pnas.org/content/97/10/5328), Copyright (2000) National Academy of Sciences, U.S.A.)

**Fig. 2:**
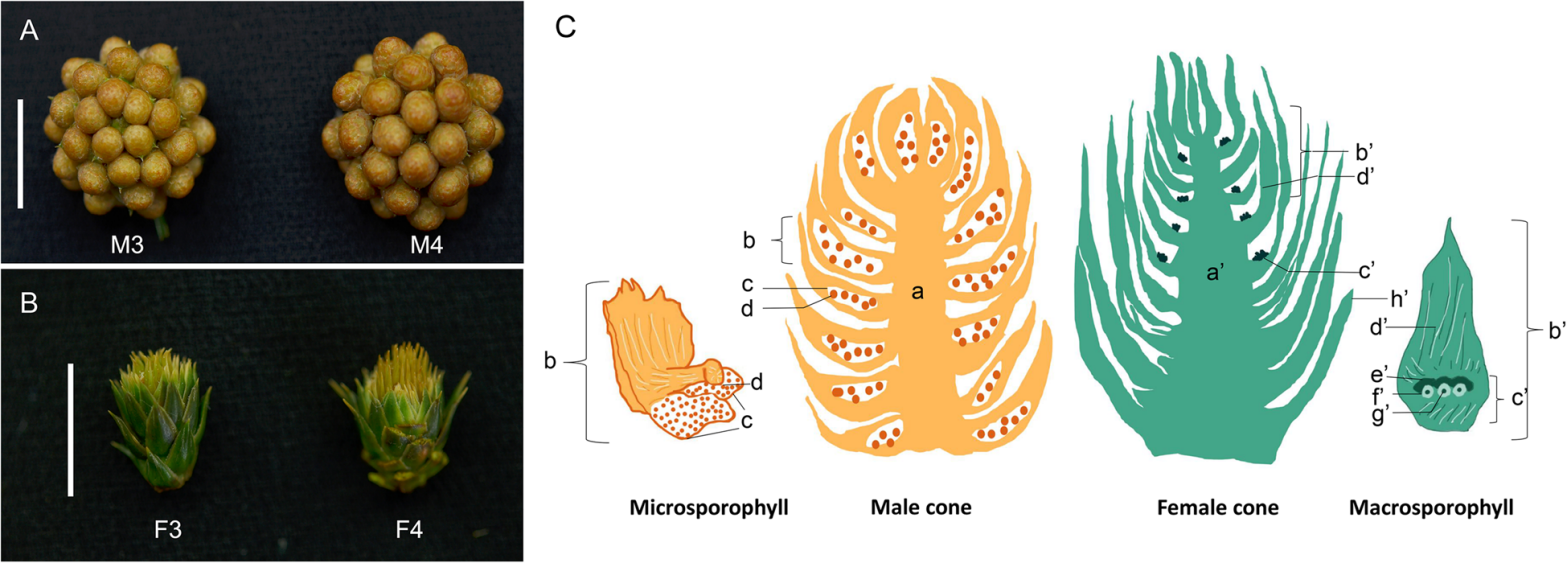
*C. lanceolata* female and male cones with their vertical section. **a** Male cone with a high number of male strobili. **b** Female cone with scale leaves and slightly opened bract-scales. Tree No.3–15-31, shown as F3, M3; Tree No.4–9-31, shown as F4, M4. Scale bar: A, B = 1 cm. **c** Male cone with axes (a), microsporophyll (b). Microsporophyll bears pollen sac (c), and pollen (d). Female cone with axes (a’), scale (h’), and macrosporophyll. Macrosporophyll with bract-scale (d’), ovuliferous scale (c’). ovuliferous scale with lobe (e’), integument (f’), nucellus (g’) [8]

*Cunninghamia lanceolata* (Lamb.) Hook (Chinese fir) is one of the most commercially important timber trees in the south of China. It has been cultivated for thousands of years because of its outstanding wood properties and high growth rate [1], with its plantation area accounting for 24% of the total plantation area in China [2]. *C. lanceolata* is a monoecious conifer species, with female cones distributed in the upper and middle crown and male cones distributed in the middle and lower crown [3]. *C. lanceolata* female/male cones differ greatly from classic angiosperm flowers (Fig. 1a). Specifically, the female cone is comprised of bract-scales with ovules produced at their base. The bract-scales will gradually open and ovules will then receive pollen to complete fertilization (Fig. 2b-c) [8]. In *C. lanceolata*, male cones are aggregated into a compound structure consisting of several strobili, each of which is wrapped by many microsporophylls that contain the pollen sac, which release pollen grains after they have matured (Fig. 2a and c) [8]. Due to its high commercial value, *C. lanceolata* is continuously improved by breeders. One of the important breeding goals is to increase its reproductive efficiency; as ~ 50–70% of its seeds may be abortive, a fundamental reason for its low germination rate [9]. Why seeds abort is not yet fully understood; two possible causes are pollen abortion and abnormal ovule development [10]. To understand the underlying processes causing seed abortion, it is therefore necessary to study the molecular mechanisms of flower/cone development.

Flower development is a complex biological process and is affected both by genetic and environmental factors [11]. In angiosperms, the classic homeotic ABC model explains how local gene expression is able to control flower identity (Class A genes, *SQUA/AP1*; Class B genes, *AP3, PI*; Class C genes, *AG*) [12]. After its initial conception, the ABC model was later expanded upon by adding class D (Class D genes, *SHP, STK*) [13] and E (Class E genes, *SEP1, 2, 3, 4*) [14] genes – the ABCDE model – as more genes were found to be involved. The model works as such that unique combinations of each homeotic gene class (A to E) that are expressed in a certain region of the developing flower, give rise to a specific flower tissue type [5]. For example, class A and E are involved in sepal formation, the combination of ABE affects petal development, BCE controls stamen formation, CE affects carpel development and DCE is involved in establishing ovule (Fig. 1a) [15, 16]. However, in gymnosperms, the development of female and male cones is assumed to be controlled by tetramers of class B and C proteins only [5, 6]. However, still two alternative perspectives on the gymnosperm floral homeotic model exist: the B(C) system (Fig. 1b) and the (A)B(C) system, in which (C) represents class C + D, (A) represents class A + E [5].

Excepting the *AP2* gene, genes belonging to the ABCDE model are members of the MADS-box gene family, which have crucial functions in floral organ development [17]. All MADS-box proteins harbor a highly conserved domain, the MADS domain, which can be grouped into two main lineages: type I (SRF-like) and type II (MEF2-like), based on sequence conservation [7]. Both MADS lineages can be found in plants, animals and fungi. However, there are some special structures, such as the K domain, that are only found in the type II MADS-box genes of plants (Fig. 1c; redrawn from Fig.1 (https://www.pnas.org/content/97/10/5328) “Copyright (2000) National Academy of Sciences, U.S.A.” )[7]. So far, type II MADS-box genes have been more thoroughly studied for their functions in plant flower development [18]. A distinguishing feature of type II MADS-box genes in plants is that they harbor three more domains than type I MADS genes: an intervening (I) domain, a keratin-like coiled-coil (K) domain, and a C-terminal (C) domain (Fig. 1c) [19]. The highly conserved MADS domain is one of the main features of this gene family, which determines DNA binding and dimerization [20]. The K domain likely mediates protein-protein interactions, and is possibly also involved in the direct interaction with other proteins [20]. The MADS domain and K domain are linked by a short intervening I domain [21]. In some MADS-box proteins the C-terminal region is involved in the transcriptional activation or ternary complex formation [22, 23]. These genes are classified as MIKC-type and are specific to plants [7].

Previous research in *C. lanceolata* has mainly focused on the regulation of cambial activity [24], EST-SSR markers development [25–27], genes associated with growth and development [28], cellulose and lignin biosynthesis [29] and proteome analysis of early seed development [1]. Until now, little is known about the molecular mechanisms of its female/male cones development. Here, we conducted an RNA-Seq transcriptomic approach to identify genes that are differentially expressed between immature female and male cones of *C. lanceolata*. This study provides a valuable resource for gymnosperm cone development-related genes and may aid in breeding trees with increased seed numbers in upcoming Chinese fir improvement programs.

## Methods

### Plant material

Immature female and male cones were collected in late February from two different living trees (No.3–15-31, No.4–9-31) that belong to a single *C. lanceolata* clone (6421 )[24]. The trees were located at the Yangkou forest station of the Chinese fir National Germplasm Bank in Fujian, China. This station has a cooperative relationship with Nanjing Forestry University. To avoid the impact of sample differences, the female/male cones were sampled at a similar state and height in the trees. The collecting state of the female cone is that covered with green scale leaves and slightly opened bracts (Fig. 2b), while the male cones are consisting of several strobili (Fig. 2a). At this time, the ovule is already appeared but not fully formed, and so as the pollen. The author Renhua Zheng was responsible for the formal identification of the samples. However, to our knowledge, there is no herbarium to deposite the voucher specimen of this specific material. For transcriptomic analysis, female/male cones were immediately frozen in liquid nitrogen and stored at − 80 °C until RNA extraction. For SEM analysis, fresh cones were collected and fixed using 2.5% glutaraldehyde (0.1 M PBS, pH 7.2). All materials were obtained with permission.

### Scanning electron microscopy

Female/male cones fixed in 2.5% glutaraldehyde were flushed with 0.1 M phosphate buffer, dehydrated using a series of graded ethanol solutions, dried using a critical point dryer (K850, EMITECH, England), mounted with double-sided adhesive tape on stubs, and coated with aurum in a sputter coater (E-1010, HITACHI, Japan). Samples were observed on a Quanta 200 scanning electron microscope (FEI, America) [30].

### RNA extraction and mRNA library construction

An ethanol precipitation protocol and CTAB-PBIOZOL reagent was used for the purification of total RNA according to the manufacturer’s instructions. Total RNA was quality controlled and quantified by a NanoDrop and Agilent 2100 bioanalyzer (Thermo Fisher Scientific, MA, USA). Oligo (dT)-attached magnetic beads were used to purify mRNA. mRNA was then fragmented, after which first- and second-strand cDNA was generated using the First Strand reaction system. Afterwards, the purified cDNA was ligated to specific adapter sequences. Then, cDNA fragments were amplified by PCR, then purified using Ampure XP Beads. An Agilent 2100 Bioanaylzer and ABI StepOnePlus Real-Time PCR System were used for quantification and quality control of the sample library. The library was then sequenced using an Illumina HiSeq 4000 platform (BGI-Shenzhen, China) (reads length 151 bp). Sequenced reads were deposited in the NCBI Sequence Read Archive (SRA) with the accession number SRR10161401, SRR10161402, SRR10161403, SRR10161404.

### Transcriptome data assembly and functional annotation

The raw data was first filtered to obtain high-quality clean data. Adapter sequences, low-quality reads (we define a low quality read as having more than 20% of its bases with a quality score below 10) and reads with more than 5% of their bases unknown were removed from the raw reads. Clean reads were then quality controlled by FastQC v0.11.7 [31]. Clean reads extended into contigs through the overlap between sequences by running Trinity (v2.0.6) [32]. Then, according to paired-end sequence information, contigs were assembled into transcript sequences.

Coding regions of assembled unigenes were annotated by mapping them to several public databases, respectively, using TransDecoder, after which a blastp algorithm [33] was run against uniprot_sprot [34] and HMMER databases with Pfam-A.hmm (Hidden Markov Model) [35] to identify conserved proteins.

Functional annotation of these sequences was performed by running blast against protein sequences from *Arabidopsis thaliana*, *Populus trichocarpa*, *Oryza sativa*, and Swiss Prot [34]. The final Gene Ontology (GO) [36] annotation result merged data from both *A. thaliana* and *P. trichocarpa*. Due to our interest in transcription factors (TFs) specifically, a gene type parameter was added to the annotation process. In all cases, the BLAST algorithm [33] was applied with an E-value parameter not greater than 10^− 5^.

### Differential expression analysis

Gene expression levels were estimated by mapping clean reads to the Trinity transcript assembly using RSEM [37] for each sample. The abundance of all genes was normalized and calculated using uniquely mapped reads via the FPKM method [38]. The software edgeR [39] was used to identify differentially expressed genes (DEGs). The resulting *P*-value thresholds were adjusted for false discovery rate (FDR) via a multiple testing approach [40]. The condition for filtering significantly differentially expressed genes (up- and down-regulated genes) was FDR < 0.01 & fold change > 2. An R package was used for visualization of results and read dispersion. Significantly DEGs were also subjected to a GO enrichment analyses through the TopGO R package [41]. To detect which transcriptional factor families were significantly enriched (P-value < 0.01) at this developmental stage, a Chi-square test was used.

### Identification of MADS-box transcription factors and MADS-box DEGs and phylogeny reconstruction

To identify *C. lanceolata* MADS-box sequences, two reported Hidden Markov Model profiles SRF (PF00319) and K-box (PF01486) were obtained from Pfam [35]. Using HMMER software [42] with these two profiles and filter condition E-value ≤1.0E-04 candidate sequences were obtained, then further verified sequences using SMART [43].

To faithfully identify differentially expressed MADS-box genes in female/male cones of *C. lanceolata*, lowly expressed MADS-box genes were removed from the DEG list by the edgeR analysis package [39], leaving 27 MADS-box unigenes. R packages pheatmap (1.0.12) and MEGA 7.0 were used to analyze expression levels and construct phylogenetic tree, which shown as heatmap clusters. Sequence raw data of one-year-old leaves [44] were downloaded from the NCBI Sequence Read Archive (SRA) database (SRX2586190) to use as a vegetative organ expression comparison.

MADS-box sequences of *A. thaliana*, *O. sativa* and *Vitis vinifera* were obtained from the Plant Transcription Factor Database (http://planttfdb.cbi.pku.edu.cn/index.php), while sequences of *Cryptomeria japonica*, *Picea abies*, *Pinus taeda* (http://congenie.org) were gained separately from three articles by Futamura et al. [45], Carlsbecker et al .[46] and Chen et al. [47]. All the reference sequences were listed in additional file 1. Subsequently, full length of multiple sequence were aligned using MAFFT [48], after which the RAxML v8.2.11 [49] was used to construct a phylogenetic tree with the PROTGAMMAAUTO mode and 100 bootstrap replications. To support phylogenetic analysis, the alignment of MADS-box genes M, I, K, C domain in *V. vinifera, P. abies*, *P. taeda*, *C. japonica*, and *C. lanceolata* were selected and showed by Texshade [50].

### qRT-PCR analysis

Several MADS-box genes were selected to validate our DEGs detection. Total RNA was obtained from immature female/male cones using a Bioteke plant total RNA extraction kit (RP3301), only replacing the lysis buffer by CTAB. Total RNA integrity was determined by gel electrophoresis (1% gel) and RNA concentration was measured using a Nanodrop-2000 spectrophotometer (Thermo, Inc.). cDNA was synthesized through a reverse transcriptase approach using the Vazyme HiScript 1st Strand cDNA Synthesis Kit(R211–02), then quantified using a Qubit 2.0 (Invitrogen). Quantitative real-time PCR (qRT-PCR) reactions were performed in triplicate using the Vazyme AceQ qPCR SYBR Green Master Mix (without ROX) (Q121–02) on a LightCycler 480 II (Roche). Gene expression analysis was performed based on three technical and biological replicates and normalized with the reference gene *CleIF3*. Expression data were calculated through the Livak calculation method, and show as log (2^-ΔΔCt^) [51].

## Results

### Female and male cones development in *C. lanceolata*

**Fig. 3:**
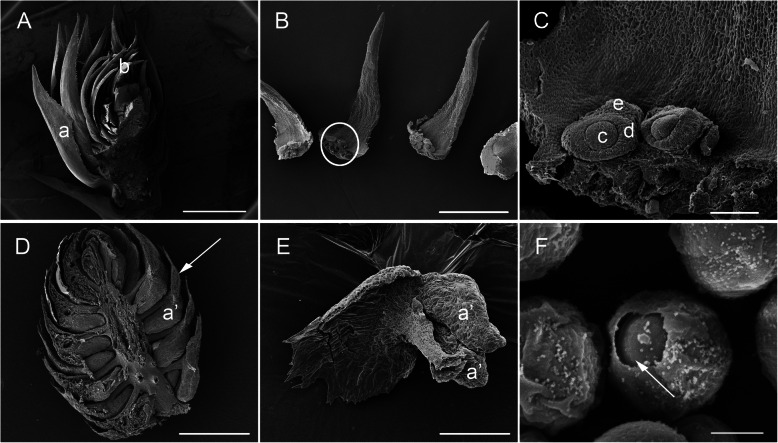
Morphology of *C. lanceolata* female and male cones. **a** Female cone with scale and macrosporophyll (b). **b** Enlarged macrosporophyll with ovule at the base of the bract-scale (ovuliferous scale). **c** Enlargement of the circle in B. Representative ovule with nucellus (c), integument (d), and lobe (e). **d** Male cone microsporophyll (indicated by arrow) bearing pollen sacs (a’). **e** Enlarged microsporophyll bearing two pollen sacs (a’). **f** Enlargement of pollen, arrow point at pollen aperture. Scale bar: A = 2 mm; B, D = 1 mm; C = 100um; E = 400um; F = 10um

Seed abortion is a non-negligible aspect of *C. lanceolata* breeding. To improve breeding values of *C. lanceolata*, it is necessary to study the molecular mechanisms of cone development. This is only exacerbated by the fact that *C. lanceolata* is a gymnosperm, and the structure of its female/male cone differs greatly from that of angiosperm flowers (Fig. 1a and Fig. 2) [52]. In order to better understand the morphological characteristics of *C. lanceolata* female/male cones, we used a Scanning Electron Microscope (SEM) to observe the female and male cones, especially to observe the ovule and pollen. At the stage we sampled, each female cone contains a high number of bract-scales, with an ovuliferous scale at the base of the bract-scale, and 2–3 ovules located at the ovuliferous scale (Fig. 3b). The lobes, nucellus, and integument (Fig. 3c) have already formed but not yet completely differentiated, one lobe of ovuliferous scale develops for each ovule (Fig. 3c) [30]. The male cone is composed of a high number of microsporophylls, one at the central position and the remaining in a surrounding spiral arrangement. Each microsporophyll bears 2–3 pollen sacs (Fig. 3d-e). Each pollen (Fig. 3f) contains a pollen aperture.

### Sequence assembly and annotation of *C. lanceolata*

We next conducted a whole transcriptomic approach to identify transcripts that are differentially regulated between the development processes of female and male cones in *C. lanceolata*. We therefore isolated total RNA from whole female/male cones and used Illumina sequencing technology to determine the transcriptome. We obtained a total of 22,188,695 (F3 female, from tree No.3–15-31), 18,114,397 (F3 male, from tree No.3–15-31), 18,731,606 (F4 female, from tree No.4–9-31) and 22,054,735 (F4 male, from tree No.4–9-31) raw reads for each library (Table S1). After filtering and removing adapter and low-quality sequences, 22,123,838 (F3 female), 18,051,760 (F3 male), 18,299,131 (F4 female) and 21,990,476 (F4 male) clean reads (Table S1) were retained for further assembly. In total, 24.14GB RNA-Seq data were generated from sequencing. We assembled a total of 97,856 transcripts with a contig N50 length of 1925 bp and 63,223 unigenes with a contig N50 length of 1721 bp (Table S2). The median contig length of all transcripts and unigenes was 784 bp and 620 bp, the average length of all transcripts and unigenes was 1228 bp and 1066 bp, respectively (Table S2). All of the 63,223 assembled unigenes (Table S2) were functionally annotated, 2117 transcription factors were identified (Table S3).

### Differential gene expression between female and male cones

**Fig. 4:**
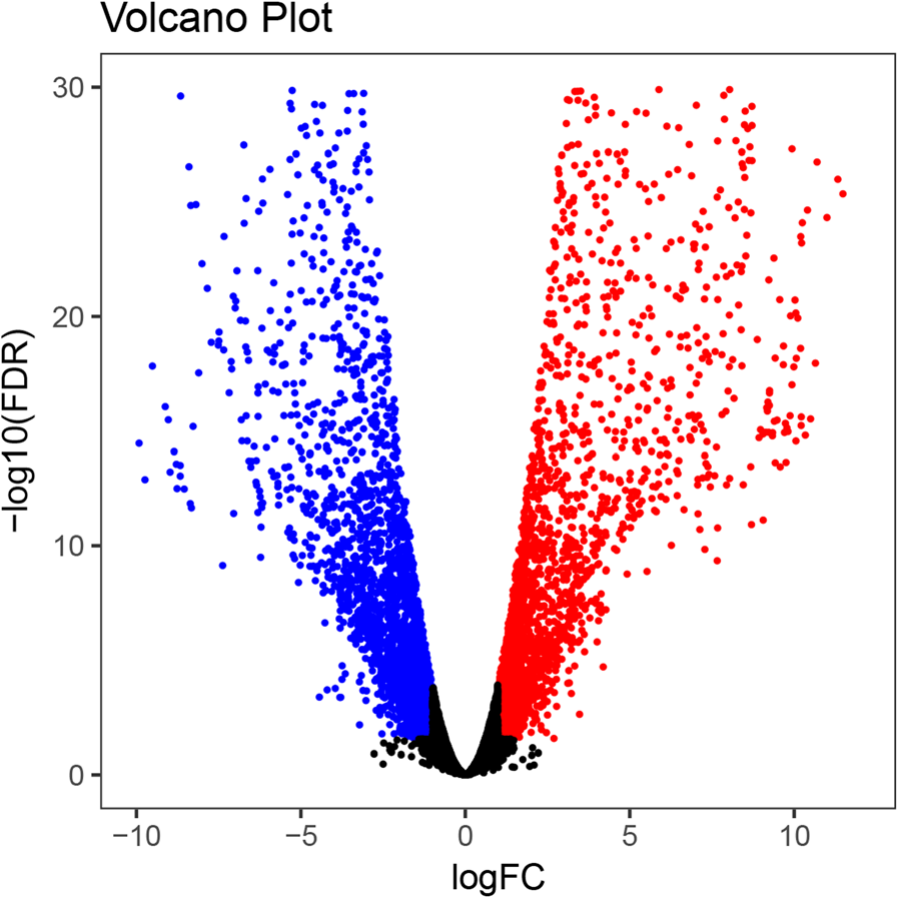
Volcano plots of differentially expressed genes. The x-axis represents the expressed fold change of genes in female and male cones. The y-axis represents the degree of statistical significance in differential expression. The higher -log10 (FDR) values represent greater differences. Black dots indicate no significant changes in gene expression. The up-regulated genes (male>female) are represented by a red dot, down-regulated (male<female) genes by a blue dot

In order to identify specific differentially expressed transcripts of *C. lanceolata* female and male cones at the same developmental stage. Among all the assembled unigenes, unigenes with low expression level were removed, resulting in 18,045 unigenes. We filtered these unigenes based on a selection criteria of FDR < 0.01 and Fold Change > 2. Then, we further characterized these genes using GO terms and functional classification. We found 5016 unigenes that were significantly differentially expressed, of which 2506 unigenes were down-regulated and 2510 unigenes were up-regulated in the male cones compared with the female cones (Fig. 4).

**Fig. 5:**
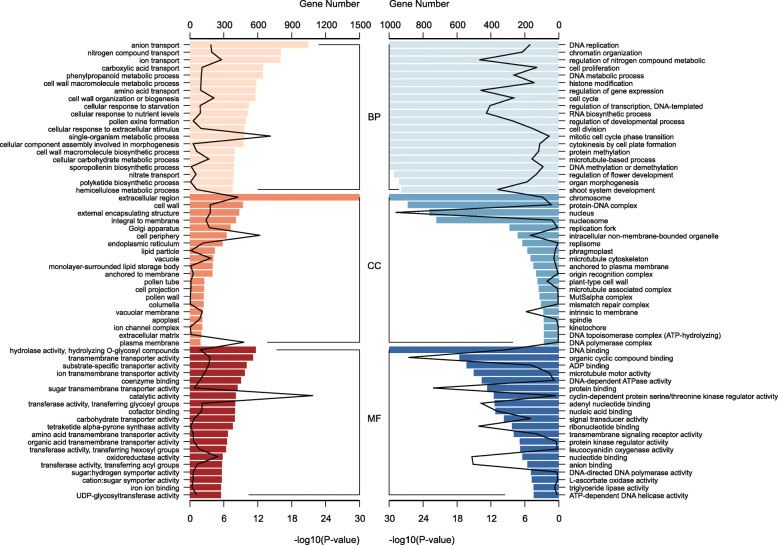
Functional classification of *C. lanceolata* DEGs. The top 20 enrichment Gene Ontology (GO) terms of three subgroups are listed. The Gene Ontology terms (GOs) were used to classify the transcript products within the category of (CC) cellular component, (MF) molecular function, and (BP) biological process sub-ontologies. The warm/cold color bars indicate the -log10 (*P*-value) of significantly expressed genes in male/female cones, while the curve represents gene number in each term

A GO enrichment analysis successfully categorized 2217/2168 of the up−/down-regulated unigenes into three GO subgroups (Fig. 5). We plotted the top 20 enriched GO terms of each subgroup, separately. The down-regulated genes (male < female, cool-toned) were involved in DNA replication (BP), nucleus (CC), protein binding (MF) etc., while the up-regulated (male > female, warm-toned) genes were involved in pollen exine formation (BP), cell wall (CC), oxidoreductase activity (MF) etc. (Fig. 5). These results indicated that cell division is active in the vigorous growth stage of female cones, while the male cone we sampled is mostly involved in pollen development.

Focusing on our previously identified 2117 *C. lanceolata* TFs, we found three gene families to be significantly enriched (*P* < 0.01): AP2, MYB-related, and MADS-box (Table 1). The significant expression of MADS-box genes during *C. lanceolata* cone development is consistent with their roles during flower formation in other plant species, suggesting that this gene family plays an important role in *C. lanceolata* as well.

**Table 1:** Summary of significantly enriched transcription factors in *C. lanceolata*

Transcription factors family	*P* value
AP2/ERF	2.28E-88
MYB-related	0.006556423
MADS-box	0.006629501

All transcription factors were calculated for significance by Chi-square test, *P* < 0.01.

### MIKC MADS-box transcription factors in *C. lanceolata*

**Fig. 6:**
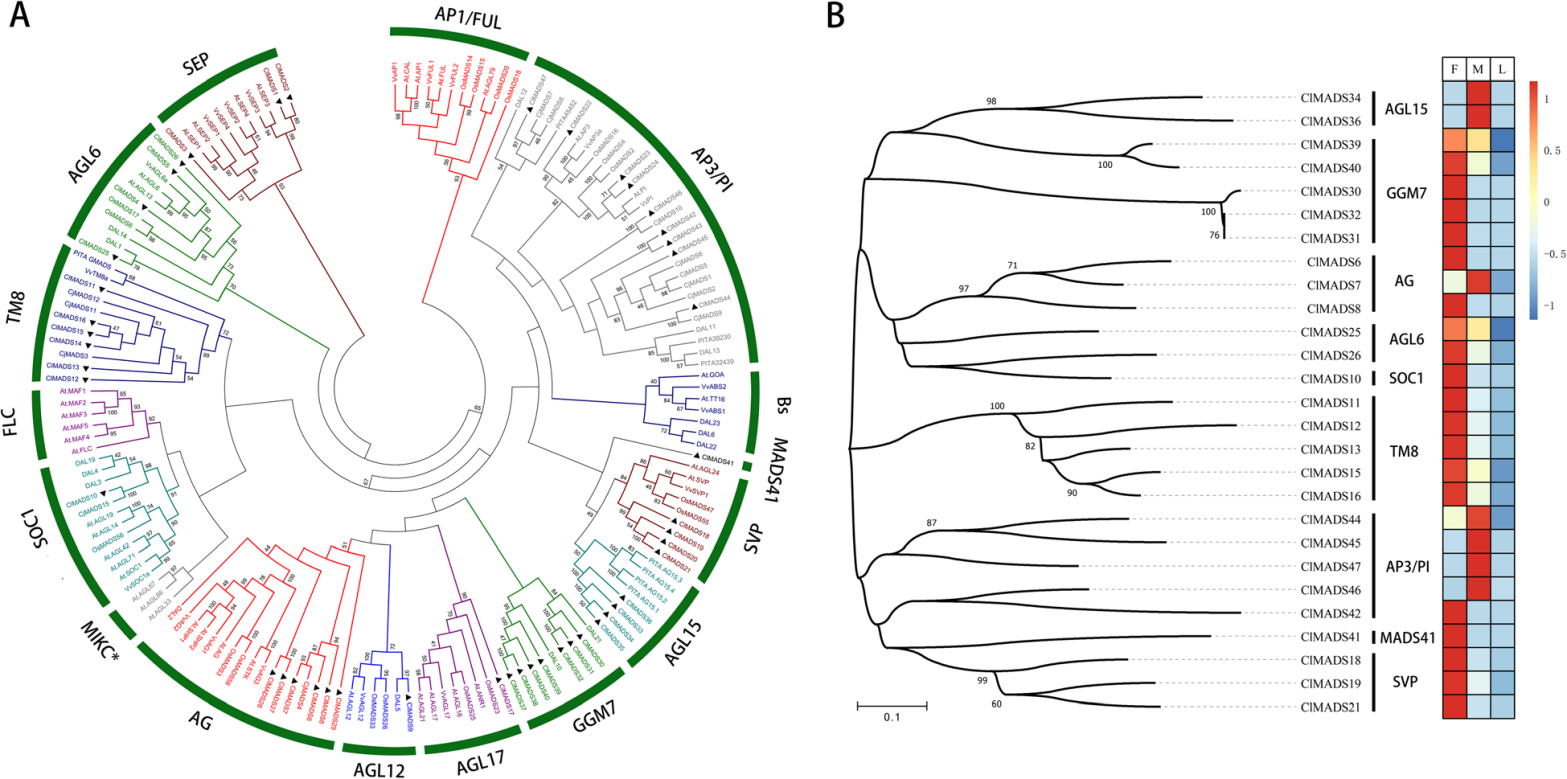
Phylogenetic tree of MIKC MADS-box genes with MADS-box DEGs in different tissues. **a** Phylogenetic analysis was performed using the Maximum Likelihood algorithm. 47 MIKC MADS-box genes were divided into 12 branches. **b** Heatmap of DEGs of MIKC MADS-box gene in female (F), male cones (M) and leaves (L)

Using the method above, we finally obtained 47 unique MIKC MADS-box genes from *C. lanceolata* (Table S4) and divided these genes into several branches, based on previous research (Fig. 6a, Tables 2 and S4) [46, 47, 53, 54]. Meanwhile, the comparison results of MADS-box proteins domain in *C. lanceolata, P. abies*, *P. taeda and V. vinifera,* making the phylogenetic analysis available (Fig. S1).

**Table 2:** Summary of MIKC MADS-box genes in *C. lanceolata*k

Type	Gene Numbers	ABCDE Class	Species
*SEP*	3	Class E	*C. lanceolata*
*AGL6*	4		*C. lanceolata*
*AG*	6	Class C & D	*C. lanceolata*
*SOC1*	1		*C. lanceolata*
*TM8*	6		*C. lanceolata*
*AGL17*	1		*C. lanceolata*
*SVP*	4		*C. lanceolata*
*AP3/PI*	9	Class B	*C. lanceolata*
*AGL12*	1		*C. lanceolata*
*GGM7*	7		*C. lanceolata*
*AGL15*	4		*C. lanceolata*
*MADS41*	1		*C. lanceolata*

Summary of genes in Fig. 6a.

Most MADS branches can be found in *C. lanceolata*, like *AP3/PI* (class B), *SEP* (class E), *AG* (class C), *STK/SHP* (class D), which are involved in flower organ identity [16]. However, branches like *FLC, BS*, *FUL* and *AP1* (class A) cannot be found. This may be explained by the low expression of these homologous genes during the selected period. Another possible reason is that the *C. lanceolata* genome does not contain these genes. Due to not having a certain typical flower structure, floral organ identity related genes, like *AP1*, which contributes to the sepal and petal formation in angiosperms, could have been lost during evolutionary time. Specific cases of such potential gene loss require further research to illustrate.

On the contrary, some MADS branches, like the *TM8* genes, are not found in *Arabidopsis* and Rice, but can be found in *C. lanceolata*, *C. japonica* [45], *V. vinifera* [53]. These results suggest that *TM8* genes were established in the common ancestor of angiosperms and gymnosperms and that they have been lost independently during the relatively recent evolution history of some plant lineages [55].

We also identified a small number of MIKC MADS-box genes that can be classified into GGM7 branches, and not found in angiosperms [46]. In contrast, *AGL15* and *AGL12* genes were found in *C. lanceolata*, and *Pinus taeda* [47], as well as in angiosperms like *A. thaliana* [56] and *V. vinifera* [53], indicating that these genes might be functionally conservative and important for both angiosperms and gymnosperms flower/cone development. Meanwhile, there is a gene that cannot be classified into any branches. We searched this gene in NCBI (https://www.ncbi.nlm.nih.gov/) using blastp and found that it was partial identity to *AG-like* gene. However, the classification cannot be gained in our phylogenetic tree. Thus, we named it with its number: *MADS41*, which make it a novel candidate gene.

### MIKC MADS-box DEGs in *C. lanceolata* female and male cones

We next used our differential gene expression data to identify which MADS-box genes are differentially expressed between female and male cones, using expression data from leaves as a comparison of non-reproductive tissue. We reasoned that genes involved in the development of reproductive organs should be more specifically expressed in those organs. Out of the 47 *C. lanceolata* MIKC MADS-box genes, 27 genes differentially expressed between male and female cones, of which 18 (out of 27) are not expressed in leaves and 9 (out of 27) are not significantly expressed in leaves. (Fig. 6b and Table S5). Most B-class genes (*AP3/PI*) (4) were up-regulated in the male cone, similar to what was found in previous studies performed in other plant species [57, 58], while *TM8* genes were clearly expressed at a higher level in the female cone and more likely to be involved in female cone development.

Since we found that some *AG* (class C + D) genes are upregulated in male cones and others in female cones, it seems likely that these genes are involved in the development of either cones. We identified three *SEP* (class E) and four *AGL6* genes in *C. lanceolata*. However, *SEP* genes showed a very low expression level, which is difficult to determine their differential expression across cones. Nevertheless, we do find two *AGL6* genes expressed in both female and male cones. In fact, during the stage we collected, *AGL6* genes showed higher expression level in the female cones.

*GGM7* genes can be subdivided into 2 categories according to the phylogenetic tree: *DAL10-like* and *DAL21-like*. They have different expression patterns in female and male cones of *P. abies* [46], as well as in *C. lanceolata*. While in *A. thaliana AGL15* expressed in leaf, inflorescences, anthers and pollen [59]; *SVP* expressed in young leaves, floral primordia and early coflorescences [60], *AGL15* and *SVP* are highly expressed in male and female cones of *C. lanceolata*, respectively. Besides, *MADS41* is a special gene with no obviously classification. But its high expression level in female cones, making it a candidate gene that may be involved in female cone development.

### Validation of the *C. lanceolata* female/male cone transcriptomes

**Fig. 7:**
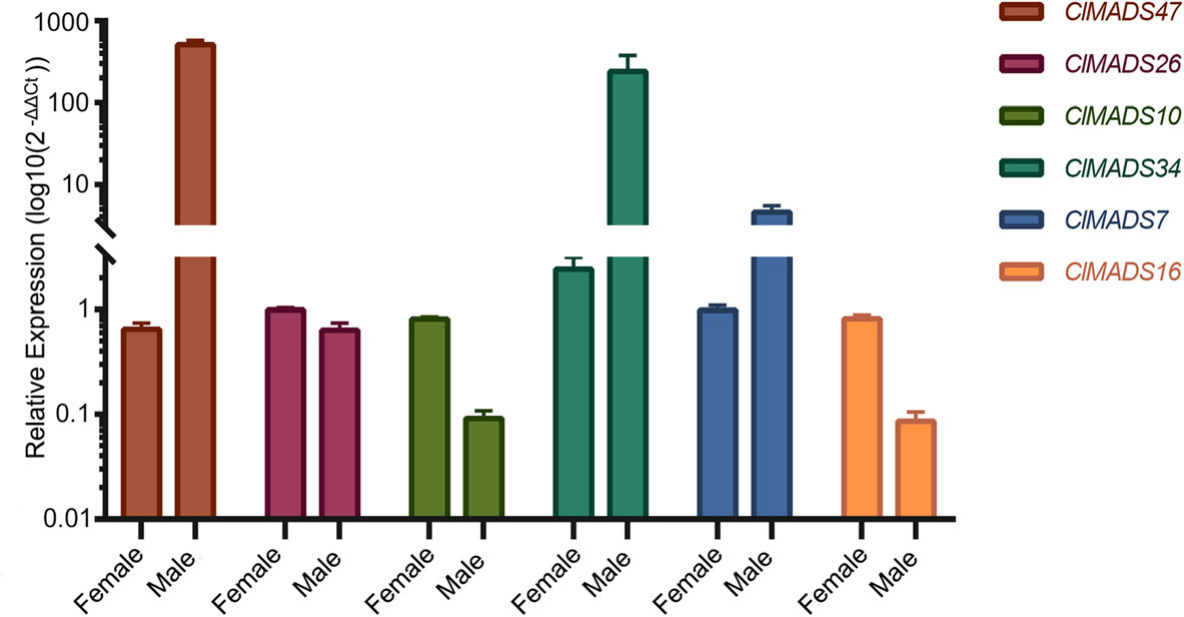
Relative expression of differentially expressed *C. lanceolata* female and male genes chosen to validate RNA-Seq results. *ClMADS7, 10, 16, 26, 34, 47* were selected for validation, *CleIF3* was used as a reference gene. The y-axis indicates the expression level (2^-ΔΔCt^), which was calculated using the Livak’s method [47] and then transformed to a log10 scale (log10 (2^-ΔΔCt^)). Error bars indicate the standard error (SE)

In order to validate the differences observed between female and male cone libraries, we selected a limited number of *C. lanceolata* MIKC MADS-box genes from the differentially expressed gene list (Table S5) and performed qRT-PCR analysis on whole cone RNA (Fig. 7). This set includes genes known to be involved in carpel or stamen development in model organisms (*AG, AP3/PI*), as well as genes not found in some angiosperms (such as *Arabidopsis* and rice) (*TM8*).

These results were in close agreement with the RNA-Seq data, for example, the expression level of *ClMADS34* gene in male cones was about 100 times that of female cones, and the expression of *ClMADS10* in female cones was almost 10 times that of male cones, which was consistent with the results of the transcriptome data, suggesting the reliability of our transcriptomic profiling data (Fig. 6b and Fig. 7).

## Discussion

As an important timber species, *C. lanceolata* reproduction has always been one of the traits sought to be improved by breeding programs. Seed abortion is a common occurrence in *C. lanceolata* and can be caused by improperly formed ovules and pollen. Here, we studied the molecular mechanisms of *C. lanceolata* cone development though a transcriptome analysis.

Based on these data, we performed sequential analyses to identify the differences between female and male cones, then we focused on the MADS-box gene family in *C. lanceolata* to reveal the potential specific genes involved in *C. lanceolata* cone development and the manifestation of the ABC model in gymnosperms.

We found class B, C, D and E genes in *C. lanceolata*, and for those genes which significantly up-regulated in male or female cone, were mostly not expressed in leaves. The B-class genes, *AP3/PI* (*ClMADS44, 45, 46, 47*) were mostly up-regulated in the male cone, which is most likely to influence male organ development. Similar results have been reported in angiosperm *Quercus suber* [57] and gymnosperm *C. japonica* [61], for example, the *CjMADS1* gene (B-type MADS-box gene), expressing in male cone through its development in *C. japonica*. As is known from Norway spruce, B-type MADS-box genes, which are active in male organ primordia [62], are homologous to the B-class genes in angiosperms [63]. These findings indicated that B-type genes are maintained in both gymnosperms and angiosperms and may be conserved throughout seed plants.

C and D-class genes cannot be separated clearly in *C. lanceolata*, and are expressed in both reproductive organs. *AG* genes expressed in both cones of *P. abies* [64] and *Gnetum gnemon*[65] (gymnosperms). This is consistent with findings in *Quercus suber* (angiosperm), where C-class genes are expressed at a similar level in both male and female flowers [57]. These results indicated that these C-type genes may play a similar role in both gymnosperms and angiosperms, which act as supply for both female and male cone/flower development [4]. Unfortunately, we were unable to identify the expression of E-class genes, as them are not significantly expressed in male or female cones. For this reason, we speculate that E-class genes are not necessary during this developmental process.

Additionally, we identified the expression of *AGL6* genes in  *C. lanceolata*, which are expressed in both female and male cones, higher in female cones, but not in leaves, similar as the expression pattern of their homologous genes in *G. gnemon* [65]. *GGM7* genes had captured great deal of attention from us since they were only found in gymnosperms [66]. In *P. abies*, the *GGM7* branch contains 2 genes: *DAL10* and *DAL21*. *DAL10* is specifically active in seed cones and pollen cones [66], and *DAL21* is not detected in male cones or vegetative shoots, but in ovuliferous scale of female cones. Meanwhlie, *ClMADS 30, 31, 32*, which were classified into *DAL21* branch, expressed in an obviously high level in female cones but not in male cones and leaves, with a similar expression pattern of *that* in *P. abies*. But things changed when it comes to *DAL10* genes. *DAL10* genes (*ClMADS 39, 40*) in *C. lanceolata* expressed in both female and male cones, and even higher in female cones. It reflects that there are both functional conservatism and functional differentiation in genes of different species.

*AGL15* and *SVP* gene act as repressor of floral transition in *A. thaliana* [59, 60], while in *C. lanceolata*, *AGL15* and *SVP* are highly expressed in male and female cones, respectively. It could be an interesting research issue and may imply a similar inhibitor in Chinese fir, restricting the development range of cones.

Furthermore, we identified several genes which may play an important role in female cone development. We detected *TM8* genes which were all up-regulated in the female cone and basically not expressed in leaves. Researchers have found that in *E. grandis*, *EgTM8* is expressed in the early and late floral bud [67]. And in tomato, *TM8* may be important for ovary and fruit formation [68]. Gramzow et al. [69] showed that *TM8* genes could be found in many gymnosperms, but little research has revealed its function in organ development in gymnosperms. Considering that ovules and pollen of *C. lanceolata* were still under development at the time of collection, we speculate that these genes are very likely to influence ovule development and can be further studied.

Based on our results, we tend to agree with the B(C) model of gymnosperm cone development proposed by Theißen et al. [5], which A and E-class genes may not involve in cone development. In order to verify the applicability of this model in *C. lanceolata*, more experiments are needed to confirm the function of B(C) genes and rule out the involvement of other genes (A, E-class genes). In a general way, we study the gene function by overexpressing and knockout this gene in the species. Unfortunately, a mature transgenic system for *C. lanceolata* has not yet been developed, and performing transgenesis experiments in this species would have the added downside that the flowering of woody plants takes a long time. Thus, other method should be considered, for example, expressed *C. lanceolata* B-type genes in model organisms such as *Arabidopsis*, so as to study the degree of functional conservation of those genes. But it must be emphasized that the gene function studies will eventually return to the species itself. Yet considering the difficulty of generating transgenic gymnosperms and their long generation times, these studies would need a lot of time and efforts.

Due to our limitation of material selection, the results were limited to the differential genes between female and male cones at a certain developmental period. Although some noteworthy genes were indeed found through our study, some information for cone development may be lost, and participation of those MADS-box genes in the entire developmental process cannot be obtained. Further research could monitor the entire developmental process, from cone initiation to female cone fertilization, to potentially find all MADS-box genes involved, and perform a more complete interpretation.

## Conclusions

In summary, we performed an RNA-Seq analysis of female and male cones in *C. lanceolata* and analyzed the gene expression differences between female and male cones. We identified 47 MIKC MADS-box genes in *C. lanceolata*, and identified some MADS-box genes related to cone development in *C. lanceolata*, possibly conforming to the previous B(C) model for gymnosperms. We also identified additional genes that may play an important role in female/male cone development. In addition, we provided a library of gene data that shows differential expression between the female and male cones, which can be used as a basis for discovering unknown regulatory networks in the future.

## Supplementary information

**Additional file 1.** All reference sequences for phylogenetic tree.

**Additional file 2 Table S1.** Sequencing statistics of *C. lanceolata* cone RNA-seq libraries. Individual libraries were generated from four specific RNA pools, two from female cones (F3 and F4) and two from male cones (M3 and M4). **Table S2.** Summary on assembled transcripts and unigenes of all samples. **Table S3.** Summary on annotation transcription factors (TFs).

**Additional file 3 Table S4.** All *C. lanceolata* MIKC type MADS-box genes.

**Additional file 4 Figure S1.** Alignment of selected MADS-box genes conserved domains. M (A), I (B), K (B & C), C (D) domain of MADS-box proteins in *V. vinifera*, *C. japonica, P. abies, P. taeda* and *C. lanceolata* (not fully length)*.*

**Additional file 5 Table S5.** Differential expressed MIKC MADS-box genes of *C. lanceolata.*

**Additional file 6 Table S6.** List of the primers used for qRT-PCR.

## Data Availability

All data generated or analyzed during this study are included in files and NCBI SRA database (Accession number SRR10161401, SRR10161402, SRR10161403, SRR10161404), and are available by contacting with the corresponding author (chenjh@njfu.edu.cn).

## Abbreviations

FPKM: Fragments per kilobase of transcript per million mapped reads
RSEM: RNA-seq by expectation maximization
edgeR: Empirical analysis of digital gene expression data in R.
