# Supplementary figures and images for: The Transcriptome of Cunninghamia lanceolata male/female cone reveal the association between MIKC MADS-box genes and reproductive organs development
Source: BMC Plant Biol. 2020 Nov 5;20:508. doi: 10.1186/s12870-020-02634-7 (PMC7643283; doi:10.1186/s12870-020-02634-7)

A

M domain

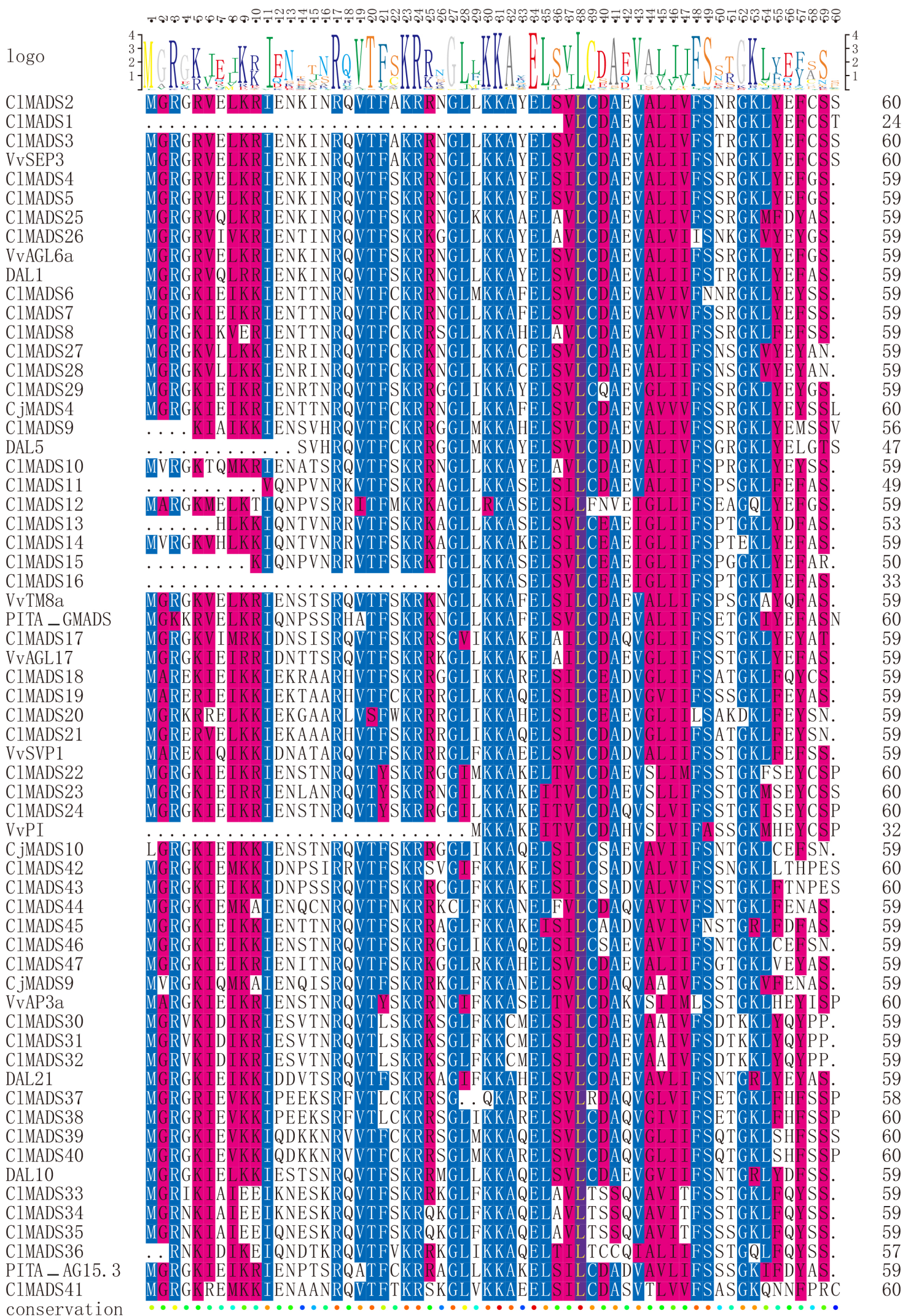

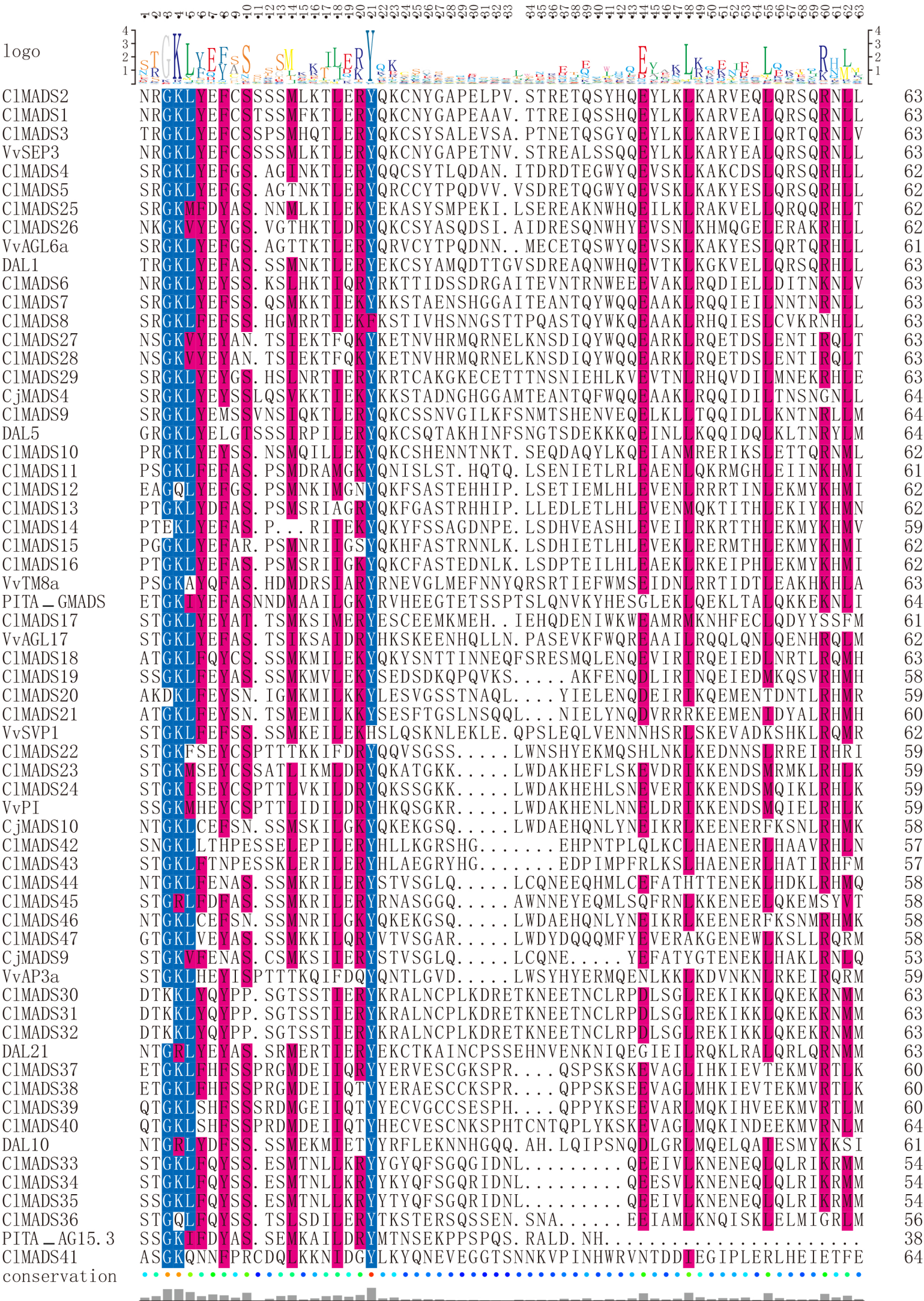

C

K domain

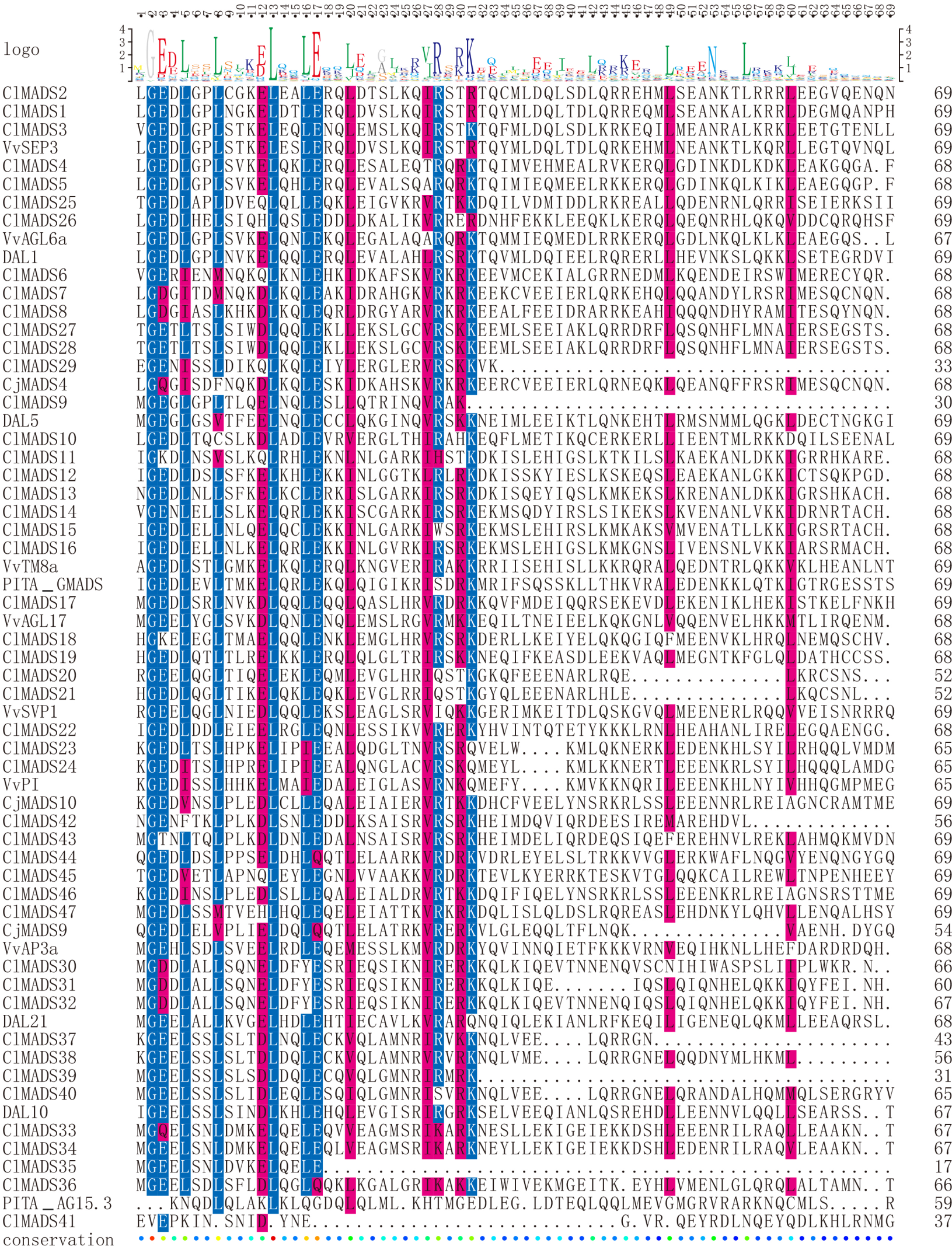

C domain

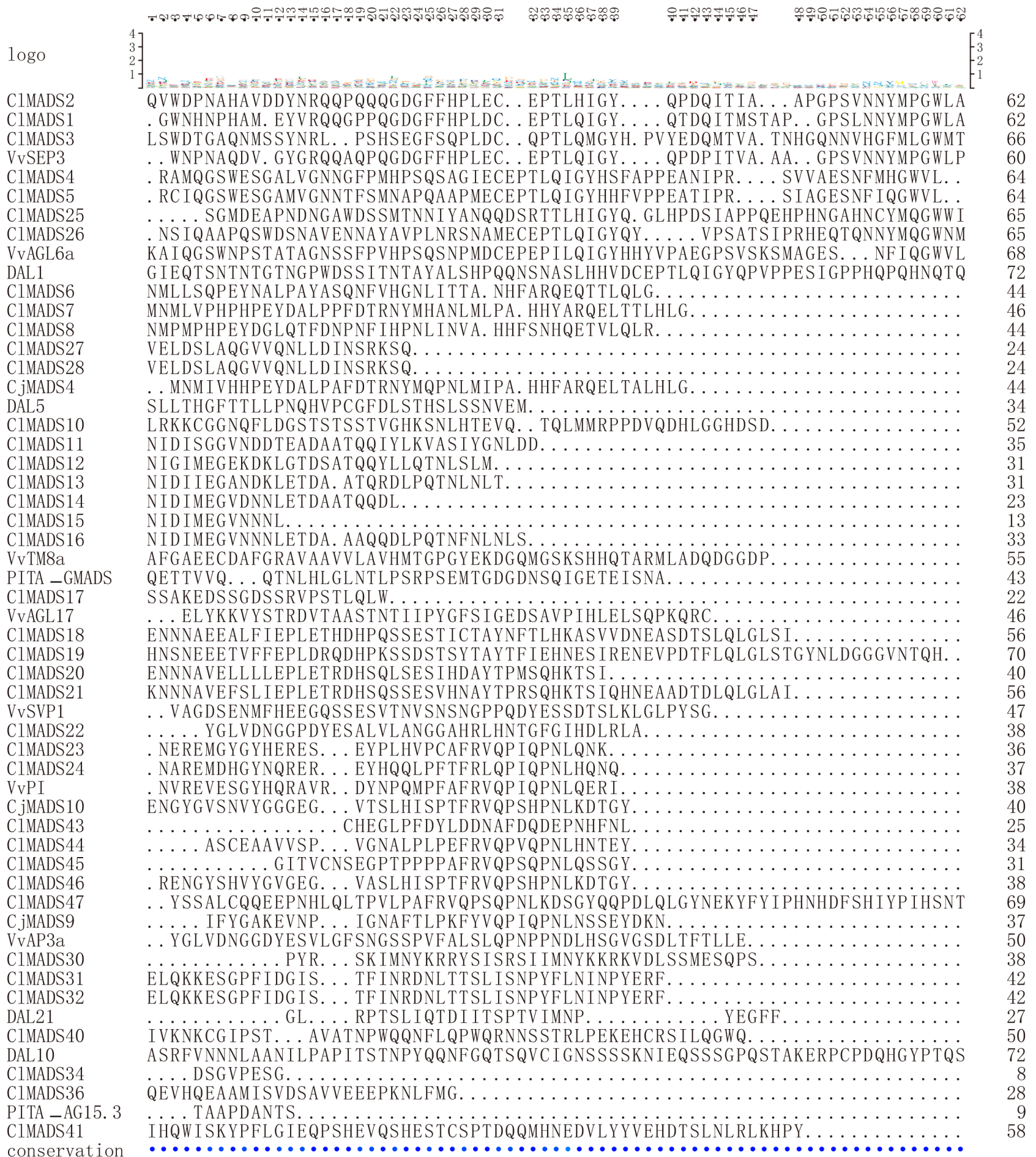

Supplement: Supplementary file 4 — Additional file 4 Figure S1. Alignment of selected MADS-box genes conserved domains. M (A), I (B), K (B & C), C (D) domain of MADS-box proteins in V. vinifera, C. japonica, P. abies, P. taeda and C. lanceolata (not fully length). [file 12870_2020_2634_MOESM4_ESM.pdf]
